# Long‐term results of a short‐term home‐based pre‐ and postoperative exercise intervention on physical recovery after colorectal cancer surgery (PHYSSURG‐C): a randomized clinical trial

**DOI:** 10.1111/codi.16860

**Published:** 2024-01-15

**Authors:** Aron Onerup, Ying Li, Kevin Afshari, Eva Angenete, Hanna de la Croix, Carolina Ehrencrona, Anette Wedin, Eva Haglind

**Affiliations:** ^1^ Department of Pediatrics, Institute of Clinical Sciences, Sahlgrenska Academy University of Gothenburg Gothenburg Sweden; ^2^ Department of Pediatric Oncology Sahlgrenska University Hospital, Region Västra Götaland Gothenburg Sweden; ^3^ School of Public health and Community Medicine, Institute of Medicine, Sahlgrenska Academy University of Gothenburg Gothenburg Sweden; ^4^ Department of Surgery, SSORG – Scandinavian Surgical Outcomes Research Group, Institute of Clinical Sciences, Sahlgrenska Academy University of Gothenburg Gothenburg Sweden; ^5^ Department of Surgery Sahlgrenska University Hospital, Region Västra Götaland Gothenburg Sweden

**Keywords:** colorectal cancer, exercise, postoperative complications, prehabilitation

## Abstract

**Aim:**

The aim of this work was to assess the effect of a short‐term, home‐based exercise intervention before and after colorectal cancer surgery on 12‐month physical recovery within a previously reported randomized control trial (RCT).

**Method:**

PHYSSURG‐C is an RCT in six participating hospitals in Sweden. Patients aged ≥20 years planned for elective colorectal cancer surgery were eligible. The intervention consisted of unsupervised moderate‐intensity physical activity 2 weeks preoperatively and 4 weeks postoperatively. Usual care was control. The primary outcome measure in PHYSSURG‐C was self‐assessed physical recovery 4 weeks postoperatively. The predefined long‐term follow‐up outcomes included: self‐assessed physical recovery 12 months postoperatively and reoperations and readmissions 91–365 days postoperatively. The statistical models were adjusted with tumour site (colon or rectum), neoadjuvant therapy (none, radiotherapy or chemo/radiotherapy) and type of surgery (open or laparoscopic).

**Results:**

A total of 616 participants were available for the 12‐month follow‐up. Groups were balanced at baseline regarding demographic and treatment variables. There was no effect from the intervention on self‐reported physical recovery [adjusted odds ratio (OR) 0.91, *p* = 0.60], the risk of reoperation (OR 0.97, *p* = 0.91) or readmission (OR 0.88, *p* = 0.58).

**Conclusion:**

The pre‐ and postoperative unsupervised moderate‐intensity exercise intervention had no effect on long‐term physical recovery after elective colorectal cancer surgery. There is still not enough evidence to support clinical guidelines on preoperative exercise to improve outcome after colorectal cancer surgery.

**What does this paper add to the literature?**

The effect of prehabilitation on clinically relevant outcome measures of long‐term physical recovery after colorectal cancer surgery is not known. We could not see any effect from an unsupervised pragmatic intervention on long‐term physical recovery. This further highlights the uncertainty of clinical benefit from prehabilitation in colorectal cancer surgery.

## INTRODUCTION

Curative treatment of colorectal cancer consists primarily of surgery, leading to a recovery process that is sometimes prolonged by postoperative complications [1]. Efforts have been made to reduce the burden of surgery by perioperative measures, including the ‘enhanced recovery after surgery (ERAS)’ concept [2]. Both preoperative physical activity and physical functional capacity [3, 4, 5, 6, 7] have been associated with postoperative recovery after colorectal cancer surgery. The levels of physical activity and fitness have also been associated with the risk of developing and mortality after a colorectal cancer diagnosis [8, 9, 10, 11, 12, 13]. This has led to several attempts to improve postoperative recovery by exercise intervention alone or in combination with other interventions [14, 15, 16, 17, 18].

A 6‐week high‐intensity exercise intervention in patients undergoing major abdominal surgery with or without cancer showed dramatic effects on postoperative complications [16]. Two intervention trials with sample sizes dimensioned for testing effects on clinically relevant outcomes could not show any effect of moderate‐intensity physical activity 2–4 weeks preoperatively in patients with colorectal cancer [14, 18]. A recent systematic review concluded that there is no evidence of any effect on postoperative complications, length of stay, readmissions or mortality in patients with colorectal cancer [19]. Two recent studies gave hope of lower complications after colorectal cancer surgery from exercise intervention, albeit limited by highly selected populations resulting in recruitment rates of 10%–21% and either a low sample size or termination at one‐third of the planned sample size with changed primary outcome [15, 17]. Even if the primary aim with prehabilitation studies is to improve postoperative recovery, the underlying hypothesis is that this could translate into long‐term benefits. Hence, long‐term follow‐up is warranted.

In this 12‐month follow‐up of PHYSSURG‐C, we aimed to assess the effect of a short‐term, home‐based exercise intervention before and after colorectal cancer surgery on long‐term physical recovery, compared with usual care.

## METHOD

### Study design

PHYSSURG‐C is a pragmatic randomized, controlled, open‐label, multicentre trial and the design has been described previously, including the full study protocol [18, 20]. Participants were recruited at five regional hospitals and one university hospital in Sweden. Ethical permission was obtained from the Regional Ethics Board in Gothenburg (2014‐10‐30, DNR:597–14). The study was registered at ClinicalTrials.gov with the trial registration number NCT02299596. The date of first registry was 17 November 2014.

### Participants

The eligibility criteria were individuals aged ≥20 years planned for elective colorectal cancer surgery at any of the recruiting hospitals. Exclusion criteria were emergency surgery, local surgery, cytoreductive surgery with subsequent hyperthermic intraperitoneal chemotherapy, inability to understand given information due to language or intellectual barriers and inability to perform study‐specific procedures (including surgery being planned too soon). Participants gave written consent and were recruited in association with their regular visits at included hospitals.

### Randomization and masking

Participants were recruited by a research nurse. After consenting to participation, participants were randomly assigned to one of the two study groups with a 1:1 allocation using a computer system creating the allocation sequence. There was a block size of four, unknown to staff. The randomization system included a screening log. Allocation was stratified according to planned surgical method (laparoscopic or open), tumour site and neoadjuvant treatment (colon, rectum without preoperative radiotherapy or rectum with preoperative radiotherapy). The system assigned participants study numbers sequentially and the study group could not be changed. The recruiting research nurse distributed baseline and follow‐up questionnaires which were returned to the study secretariat by post. Before inclusion, all participants received general information regarding the aim of the study; this contained no specific information on type or dose of physical activity to reduce the risk of contamination of the control group. Neither participants nor staff were blinded to group allocation, while research staff reviewing postoperative complications and length of hospital stay were masked regarding allocation.

### Procedures

Participants in the control group received usual care both pre‐ and postoperatively. Participants in the intervention group met individually with a physiotherapist, when the participants' previous experiences and barriers to physical activity were explored and types of aerobic physical activity were discussed [21]. They also received written and oral information regarding the intervention, consisting of two elements to be performed 14 ± 4 days preoperatively, to align with the time frames stipulated in the standardized care pathways for colorectal cancer in Sweden:

1. Thirty minutes of daily aerobic activity added to the individual's normal physical activity routine. This element was individualized in terms of type of aerobic activity and where the intervention was performed. The intensity of the activity was instructed to be of relative medium‐intensity activity according to Borg's rating of perceived exertion scale [22].
2. Inspiratory muscle training. The participant's maximal inspiratory pressure was determined at residual volume with a MicroRPM respiratory pressure meter (CareFusion, Höchberg, Germany) [23]. The participants were instructed to perform inspiratory muscle training with 30 × 2 breaths, twice daily, starting with a resistance of 30% of maximal inspiratory pressure (Philips Respironics, Eindhoven, The Netherlands). The participants were instructed on how to increase the resistance.

The postoperative intervention started when participants were discharged from the hospital. Participants in the intervention group were instructed to resume the same dose and intensity of aerobic activity as during the preoperative intervention and to continue for 4 weeks. No inspiratory muscle training was to be performed postoperatively.

Participants in the intervention group were contacted by telephone by the research nurse 1 week into the preoperative intervention and 3 weeks into the postoperative intervention. This included follow‐up of the intervention and allowed for modifications as needed. Adverse events related to the intervention were also registered by the research nurse in the follow‐up phone calls.

During hospitalization, participants in both groups received the same information on the importance of early mobilization postoperatively and were instructed to use deep breathing exercises hourly with positive expiratory pressure according to local routine. Local routines for pre‐ and postoperative care, including varying degrees of adherence to the ERAS protocol, were followed.

### Data collection

The research nurse registered baseline demographic information at inclusion. Participants were asked to complete questionnaires at inclusion and 4 weeks and 12 months postoperatively. The baseline level of physical activity was assessed with the Saltin–Grimby physical activity level scale [24]. Two investigators, masked regarding allocation, collected information on postoperative complications, length of hospital stay, readmission and reoperations from healthcare records at the hospitals performing the index surgery.

### Outcomes

The primary outcome in PHYSSURG‐C was self‐assessed physical recovery 4 weeks postoperatively, and the results have been published together with other measures of physical recovery within 90 days postoperatively [18]. For a full list of the outcomes in the study, see the published protocol [20].

The secondary outcomes reported in this 12‐month follow‐up were:

1. Self‐reported physical recovery 12 months postoperatively. Assessed with the question ‘To what extent do you feel fully physically recovered?’, with answering categories not recovered, 25%, 50%, 75% and fully recovered [6, 25].
2. Reoperation 91–365 days postoperatively. Defined as all types of surgery under general anaesthesia performed that could be related to the colorectal cancer surgery or the oncological treatment, between days 91–365 after primary surgery.
3. Readmissions. All re‐admissions 91–365 days after surgery that could be related to the index surgery or treatment were registered.

### Statistical analysis

As described previously, the sample size was estimated for the primary outcome at 4 weeks postoperatively [18, 20]. The statistical analyses were performed according to a prespecified statistical analysis plan. Self‐reported physical recovery was analysed with a proportional odds logistic regression model, with the result presented as an odds ratio (OR). Reoperations and readmittances were analysed with logistic regression models. Analyses were performed both in the intention‐to‐treat population as well as in the per protocol population, where activity ≥8 days preoperatively according to the exercise diary was considered as adherence to the intervention. Missing values were handled by a listwise principle.

Statistical analyses were performed using R, version 4.2.2. The proportional odds logistic model was estimated using the MASS package.

## RESULTS

Between 22 January 2015 and 28 May 2020, 761 participants were randomized to intervention (*n* = 379) or control (*n* = 382; Figure 1). The study stopped at full accrual. At 12 months postoperatively, 616 (81%) participants were available for follow‐up of reoperations and readmissions, of whom 518 (84%) contributed with self‐reported physical recovery. Mean age was 68 years and 39% of participants were female. Participants in the intervention and control groups were balanced at baseline regarding age, gender, education, ethnicity, occupational status, smoking, risk drinking, baseline level of physical activity, body mass index, comorbidity, tumour stage, type of surgery and American Society of Anesthesiologists (ASA) classification (Table 1). A total of 192 (67%) of the 287 participants in the intervention group registered activity ≥8 days preoperatively and were included in the intervention group in the per protocol analysis. There were no reported adverse events related to the intervention.

**FIGURE 1:**
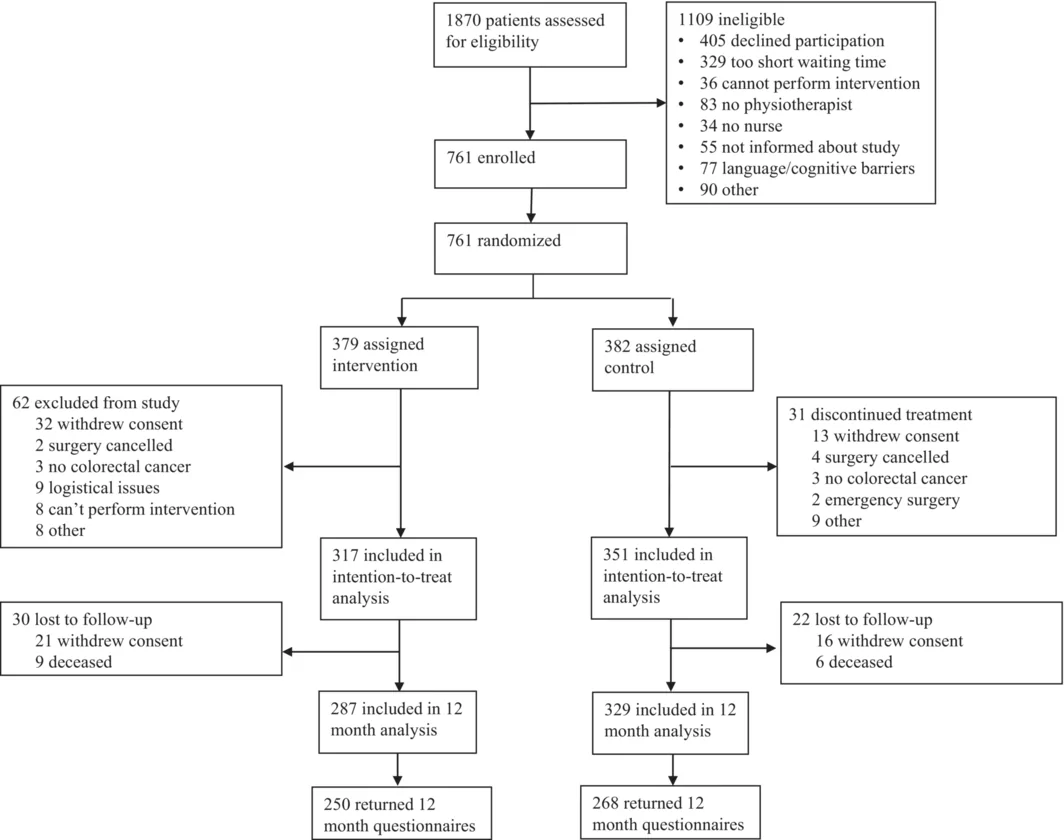
Flowchart of study cohort.

**TABLE 1:** Baseline demographic characters.

Variable	Control (*n* = 329)	Intervention (*n* = 287)
Age (years), mean (SD)	68.0 (11.6)	67.5 (10.9)
Body mass index (kg/m^2^), mean (SD)	25.9 (4.3)	25.9 (3.9)
Missing, *n* (%)	3 (1%)	0 (0%)
Female gender	129 (39%)	112 (39%)
Level of education, *n* (%)		
Compulsory school	67 (20%)	53 (18%)
High school	136 (41%)	115 (40%)
University	107 (33%)	102 (36%)
Missing	19 (5.8%)	17 (5.9%)
Ethnicity, *n* (%)		
Born abroad	41 (12%)	40 (14%)
Missing	18 (5.5%)	12 (4.2%)
Occupation, *n* (%)		
Retired	194 (59%)	168 (59%)
Sick leave/unemployed	31 (9.4%)	18 (6.3%)
Working	87 (26%)	85 (30%)
Missing	17 (5.2%)	16 (5.6%)
Smoking, *n* (%)		
No active regular smoking	303 (92%)	265 (92%)
Missing	18 (5.5%)	14 (4.9%)
Risk drinking, *n* (%)	46 (14%)	40 (14%)
Missing	73 (22%)	51 (18%)
Level of physical activity at baseline, *n* (%)		
Sedentary	55 (17%)	43 (15%)
Some physical activity	203 (62%)	183 (64%)
Regular physical activity and training	43 (13%)	43 (15%)
Regular hard physical training	5 (1.5%)	2 (0.7%)
Missing	23 (7%)	16 (5.6%)
Level of physical activity 12 months postoperatively, *n* (%)		
Sedentary	45 (14%)	38 (13%)
Some physical activity	162 (49%)	164 (57%)
Regular physical activity and training	54 (16%)	42 (15%)
Regular hard physical training	3 (0.91%)	2 (0.7%)
Missing	65 (20%)	41 (14%)
Comorbidity, *n* (%)		
Cardiovascular disease	120 (36%)	111 (39%)
Diabetes mellitus	38 (12%)	38 (13%)
Pulmonary disease	11 (3.3%)	10 (3.5%)
Mental disease	27 (8.2%)	20 (7%)
Neurological disease	5 (1.5%)	6 (2.1%)
Other disease	130 (40%)	102 (36%)
Tumour stage, *n* (%)		
0	1 (0.3%)	1 (0.35%)
I	86 (26%)	78 (27%)
II	91 (28%)	65 (23%)
III	100 (30%)	104 (36%)
IV	24 (7.3%)	16 (5.6%)
Missing	27 (8.2%)	23 (8%)
ASA classification, *n* (%)		
1	57 (17%)	51 (18%)
2	183 (56%)	164 (57%)
3	62 (19%)	47 (16%)
4	0 (0%)	2 (0.7%)
Missing	27 (8.2%)	23 (8%)
Type of surgery, *n* (%)		
Colon resection	132 (40%)	132 (46%)
Lower anterior resection	94 (29%)	69 (24%)
Rectum amputation	65 (20%)	54 (19%)
Missing	38 (12%)	32 (11%)

*Abbreviation*: ASA, American Society of Anesthesiologists.

At 12 months postoperatively, 47% of participants reported that they were fully physically recovered (Table 2) compared with 14% at 4 weeks postoperatively [18]. There were no differences between participants in the intervention and control groups, neither in the intention‐to‐treat analysis (OR 0.91, *p* = 0.60; Table 3) nor in the per protocol analysis (OR 1.12, *p* = 0.56; Table 3).

**TABLE 2:** Descriptive information about outcomes for the intention to treat population.

Outcome	Control (*n* = 244 for self‐reported physical recovery, *n* = 329 for other outcomes)	Intervention (*n* = 262 for self‐reported physical recovery, *n* = 287 for other outcomes)
Self‐reported physical recovery 12 months postoperatively		
Not applicable	7 (2.7%)	7 (2.9%)
25% recovered	10 (3.8%)	11 (4.5%)
50% recovered	24 (9.2%)	25 (10%)
75% recovered	95 (36%)	87 (36%)
Fully recovered	126 (48%)	114 (47%)
Length of hospital stay (days) 91–365 days postoperatively (median, 75th percentile, 95th percentile	0, 0, 5.6	0, 0, 10
Hospital readmission 91–365 days postoperatively	53 (16%)	44 (15%)
Reoperation 91–365 days postoperatively	31 (9.4%)	25 (8.7%)
Number of reoperations 91–365 days postoperatively		
0	298 (91%)	262 (91%)
1	27 (8%)	21 (7%)
2	4 (1%)	3 (1%)
3	0	1 (0.3%)

**TABLE 3:** Adjusted analyses for intention to treat (*n* = 616) and per protocol (*n* = 521) analyses.

Risk factor	Odds ratio for intervention versus control	95% confidence interval	*p*
Self‐reported physical recovery 12 months postoperatively			
Intention‐to‐treat	0.91	(0.65, 1.28)	0.60
Per protocol	1.12	(0.77, 1.63)	0.56
Reoperation 91–365 days			
Intention‐to‐treat	0.97	(0.54, 1.72)	0.91
Per protocol	1.07	(0.55, 2.01)	0.84
Re‐admission 91–365 days			
Intention‐to‐treat	0.88	(0.55, 1.39)	0.58
Per protocol	0.80	(0.46, 1.35)	0.41

*Note*: The models were adjusted for tumour site (colon or rectum), neoadjuvant therapy (none, radiotherapy or chemo/radiotherapy) and type of surgery (open or laparoscopic). The missing values were handled on a listwise principle.

During the 91–365 days postoperative time frame, 97 (16%) of the participants were admitted to hospital for causes that could be related to the index surgery or treatment (Table 2). This did not differ between the intervention and control groups (OR 0.88, *p* = 0.58 for the intention‐to‐treat analysis; OR 0.80, *p* = 0.41 for the per protocol analysis; Table 3). During the same time frame, 31 (9.4%) of participants in the control group and 25 (8.7%) of participants in the intervention group underwent at least one reoperation, with a majority undergoing one reoperation (Table 2). This did not differ between the groups in our multivariable analyses (OR 0.97, *p* = 0.91 for intention‐to treat analysis; OR 1.07, *p* = 0.84 for per protocol analysis; Table 3).

## DISCUSSION

This home‐based unsupervised moderate intensity exercise intervention 2 weeks before and 4 weeks after curative surgery for colorectal cancer did not improve long‐term physical recovery or reduce the risk of reoperation or readmission. This applied both to the intention‐to‐treat analysis and to the per protocol analysis restricted to those who reported adherence to the intervention.

This is in accordance with a recent systematic review: preoperative interventions including at least nutrition and physical activity appear to improve physical performance and quality of life but not postoperative complications, hospital length of stay, hospital readmissions or mortality in patients with colorectal cancer [19]. There are relatively few randomized controlled trials (RCTs) designed to evaluate possible effects of prehabilitation, including (but not limited to) exercise, on clinically relevant measures of postoperative recovery after colorectal cancer. Carli et al. reported no effect on postoperative complications from a 4‐week supervised and personalized preoperative moderate‐intensity exercise intervention in 110 frail participants with colorectal cancer [14]. We reported no effect from a 2‐week preoperative and 4‐week postoperative unsupervised moderate‐intensity exercise intervention on short‐term self‐reported physical recovery, postoperative complications, length of hospital stay, reoperation or readmission [18]. A smaller study from the Netherlands selected 57 high‐risk patients with low fitness and randomized them to usual care or a 3‐week supervised moderate‐ to high‐intensity exercise intervention [17]. They concluded that the exercise intervention reduced postoperative complications in high‐risk patients. However, participants were unbalanced at baseline, with more smokers, a higher comorbidity load and more open surgery in participants in the control group. Furthermore, they reported differences in the number of patients with any postoperative complication but no difference in the comprehensive complications index, length of stay or reoperations. A recent international study in 251 participants with colorectal cancer reported a ≈50% lower risk of a comprehensive complications index ≥20 (effectively a complication requiring medication or more intensive interventions) following 4 weeks of supervised high‐intensity exercise [15]. However, the population was selected with a recruitment rate of 22%, and the study was terminated at 35% of planned accrual due to the COVID pandemic and the primary outcome was changed. Hence, it remains to be shown whether these results could be repeated in an RCT reaching full power to show an effect on clinically relevant outcomes. Furthermore, those results only apply to surgical high‐risk patients with colorectal cancer (11% of the population) and cannot be extrapolated to the full colorectal cancer population. Still, these results are partly in line with the impressive reduction of postoperative complications seen in a Spanish study where patients scheduled to undergo major abdominal surgery, including but not limited to cancer, were randomized to a personalized, supervised, 6‐week high‐intensity exercise intervention [16]. They reported a 50% reduction in postoperative complications. Combined, these results indicate that high‐intensity training for a longer duration than what is currently possible within most national cancer pathway guidelines may be effective. The Dutch study prolonged time to surgery for participants in the intervention group to give time for the intervention [17], and we have recently reported that this should be safe in colon cancer patients, based on analyses in a population‐based sample [26].

To our knowledge, none of the other large RCTs have reported long‐term follow‐up of their interventions. However, Trépanier et al. reported no difference in overall or disease‐free survival in the full population but a higher disease‐free survival following prehabilitation in participants with Stage III colorectal cancer in a pooled analysis of three smaller RCTs [27]. While this may occur due to multiple comparisons from several reanalyses and combinations of small RCTs, it shows a possible long‐term potential of prehabilitation that should be further examined in existing larger RCTs. It is also in line with data on physical activity/fitness and survival after colorectal cancer diagnoses from observational studies [9, 11, 12, 13].

This study has several strengths. One is the large sample size, allowing for sufficient power to detect clinically meaningful effects. Another is the study design, with wide inclusion criteria and an intervention that is feasible to implement in clinical practice. The fact that the secondary outcome measures analysed in this manuscript were preselected at the outset of the study reduces the risk of data fishing. The exercise intervention in PHYSSURG‐C was designed to align with general recommendations on physical activity, allowing for other health benefits than strictly postoperative ones [10]. Furthermore, with a cancer diagnosis being a life‐defining event, the addition of a postoperative intervention aimed at long‐term behavioural changes during survivorship is a strength. However, the combined short‐ and long‐term results from PHYSSURG‐C indicate that the short‐term unsupervised moderate‐intensity exercise intervention was not sufficient to improve measures of postoperative physical recovery. This is a limitation, and the current combined evidence favours high‐intensity exercise for 4–6 weeks for the next large‐scale RCT. However, we consider it a strength that PHYSSURG‐C has evaluated the effect of general recommendations on the effects of prehabilitation in an unselected colorectal cancer population, and that we can state with relative certainty that they do not have any effect of relevance. Another limitation is the relatively low recruitment rate (41%) which reduces external validity. However, the other three RCTs in colorectal cancer populations have reported recruitment rates of 29% [14], 22% [15] or 11% [17], reflecting highly selected populations and even more limited external validity. Taken together, our and other recent trials indicate a substantial difficulty in introducing physical activity programmes to patients diagnosed with colorectal cancer. In our experience, inviting patients with colorectal cancer to participate in trials of new interventions would result in recruitment of somewhere around 50%. A limitation with the current study is that we only report secondary outcome measures and that the sample size calculation was not performed for any of these outcomes at this length of follow‐up.

These results further highlight the need for continued research on prehabilitation in colorectal cancer surgery before general recommendations are made. Currently, the ERAS guidelines for perioperative care in elective colorectal cancer surgery from 2018 include a weak recommendation on prehabilitation based on low‐quality evidence of benefits for postoperative recovery [2]. While there are a couple of promising studies [16, 28], the only other high‐quality study in colorectal patients reaching full accrual also showed no effect [14]. We consider it reasonable to believe that the current and previous results from PHYSSURG‐C describe the effect to be expected from general recommendations on unsupervised preoperative physical activity [18]. Unsupervised exercise isn't costly, has several other known health benefits and should be recommended during long‐term disease‐free cancer survivorship [29]. However, the preoperative time frame, where the patient has received a cancer diagnosis and is adapting emotionally, is challenging, and exercise recommendations should be evidence‐based and cost‐effective. If implemented in clinical practice, we think current evidence only supports supervised high‐intensity exercise for a minimum of 4 weeks [15, 16].

To conclude, a pre‐ and postoperative unsupervised moderate‐intensity exercise intervention had no effect on short‐ or long‐term physical recovery after elective colorectal cancer surgery. There is still not enough evidence to support clinical guidelines on preoperative exercise to improve outcome after colorectal cancer surgery.

## AUTHOR CONTRIBUTIONS

**Aron Onerup:** Conceptualization; investigation; funding acquisition; writing – original draft; methodology; writing – review and editing; data curation. **Ying Li:** Writing – review and editing; methodology; investigation; formal analysis. **Kevin Afshari:** Methodology; writing – review and editing; investigation. **Eva Angenete:** Conceptualization; investigation; methodology; writing – review and editing; project administration; supervision. **Hanna de la Croix:** Conceptualization; funding acquisition; investigation; methodology; writing – review and editing. **Carolina Ehrencrona:** Methodology; writing – review and editing; data curation. **Anette Wedin:** Investigation; writing – review and editing; methodology; project administration. **Eva Haglind:** Conceptualization; investigation; funding acquisition; methodology; writing – review and editing; project administration; supervision; resources.

## FUNDING INFORMATION

This study was funded by grants from AFA insurance (150072), the Swedish state under the agreement between the Swedish government and the county councils, the ALF‐agreement (ALFGBG‐718221, ALFGBG‐4307771, ALFGBG‐493341 and ALFGBG‐784821), the Swedish Cancer Society (CAN 2016/519; 2019/190303), Assar Gabrielsson's foundation (FB19‐07 and FB 16–95), Anna‐Lisa and Bror Björnsson's Foundation, Dr Felix Neubergh's Foundation (2017–347), Gothenburg Medical Society (GLS‐688001, GLS‐778731 and GLS‐883991), Lions Cancer Fund West (2019:6), Mary von Sydow's Foundation (1216), the Swedish Society of Medicine (SLS‐499811) and the healthcare committee Region Västra Götaland (Hälso‐ och sjukvårdsstyrelsen) (VGFOUREG‐309261, VGRFOUREG‐659011, VGRFOUREG‐837542, VGRFOUREG‐739191). The funders of the study had no role in study design, data collection, data analysis, data interpretation or writing of the report. The corresponding author had full access to all the data in the study and had final responsibility for the decision to submit for publication.

## CONFLICT OF INTEREST STATEMENT

All authors have completed the ICMJE uniform disclosure form at www.icmje.org/coi_disclosure.pdf and declare: EA, CE, HdlC and AW declare no support from any organization for the submitted work; AO reports grants from the Swedish state under the agreement between the Swedish government and the county councils, the ALF agreement, from Assar Gabrielsson's foundation, Anna‐Lisa and Bror Björnsson's foundation, Dr Felix Neubergh's foundation, Gothenburg Medical Society, and Lions Cancer Fund West during the conduct of the study; EH reports grants from AFA insurance and grants from the Swedish state under the agreement between the Swedish government and the county councils, the ALF‐agreement, and grants from The Swedish Cancer Society during the conduct of the study; AO, EA, CE, HdlC and AW declare no financial relationships with any organizations that might have an interest in the submitted work in the previous 3 years; EH reports grants from the Mary von Sydow Foundation, grants from the Swedish Research Council and grants from Göteborg Medical Society outside the submitted work; all authors report no other relationships or activities that could appear to have influenced the submitted work. All authors confirm that they had full access to all the data in the study and accept responsibility to submit for publication.

## DATA SHARING

Individual participant data that underlie the results reported in this article, after de‐identification (text, tables, figures and appendices), as well as the study protocol, statistical analysis plan and analytic code will be shared with investigators whose proposed use of the data has been approved by an independent review committee (‘learned intermediary’) identified for this purpose, and after assuring that this data‐sharing complies with ethical permissions. Data may be used for individual participant data meta‐analysis. Data sharing proposals should be sent to the corresponding author.

## ETHICS STATEMENT

Ethical permission was obtained from the Regional Ethics Board in Gothenburg (2014‐10‐30, DNR:597‐14).

## CONSENT

Participants gave written consent and were recruited in association with their regular visits at included hospitals.

## Data Availability

The data that support the findings of this study are available on request from the corresponding author. The data are not publicly available due to privacy or ethical restrictions.
